# Spatial distribution and determinants of newborn care within 2 days of postpartum period among women with at least one antenatal care visit in Ethiopia: Mixed effect multilevel analysis

**DOI:** 10.1371/journal.pone.0282012

**Published:** 2023-02-28

**Authors:** Melaku Hunie Asratie, Daniel Gashaneh Belay, Belayneh Ayanaw Kassie, Nuhamin Tesfa tsega, Fantu Mamo Aragaw, Moges Gashaw, Mastewal Endalew

**Affiliations:** 1 Department of Women’s and Family Health, School of Midwifery, College of Medicine and Health Sciences, University of Gondar, Gondar, Ethiopia; 2 Department of Human Anatomy, College of Medicine and Health Sciences, University of Gondar, Gondar, Ethiopia; 3 Department of Epidemiology and Biostatistics, Institute of Public Health, College of Medicine and Health sciences, University of Gondar, Gondar, Ethiopia; 4 Department of Physiotherapy, School of Medicine, College of Medicine and Health sciences, University of Gondar, Gondar, Ethiopia; 5 Department of Environmental and Occupational Health, and Safety, Institute of Public Health, College of Medicine and Health sciences, University of Gondar, Gondar, Ethiopia; JSI Research and Training Institute Inc, ETHIOPIA

## Abstract

**Introduction:**

Neonatal mortality is pervasive in developing countries like Ethiopia. Though the risk of neonatal mortality is preventable through consolidating simple, low-cost, and less time-consuming essential care, there is a scarcity of evidence about the spatial distribution of newborn care in Ethiopia.

**Objective:**

The current study aimed to demonstrate spatial distribution and determinants of newborn care within 2 days of the postpartum period in Ethiopia.

**Methods:**

A cross-sectional study was employed based on Ethiopian demographic and health survey 2016 data and 2796 post-partum period women were included. Arc GIS version 10.7 and SaTScan version 9.6 software were used. Mixed effect analysis was done by STATA version 14 software. Bivariate analysis was done and variables with a p value<0.2 were taken as a candidate for multilevel multivariable logistic regression. Intra Class Correlation Coefficient (ICC), Proportion Change in Variance (PCV), and Median Odds Ratio (MOR) were used for model comparison and an Adjusted Odds Ratio (AOR) with respect to a 95% confidence interval was used for declaring statistical significance. In the multivariable analysis, a p-value≤0.05 was considered as a cut point of statistical significance with the outcome variable.

**Results:**

The spatial distribution of newborn care was not random and the overall prevalence was 48.39%. Secondary educational level (Adjusted Odds Ratio (AOR = 1.5;95% CI 1.06,2.62), college and above (AOR = 2.47; 95% CI 1.22,5.01), number of antenatal cares three (AOR = 1.5; 95% CI 1.10, 2.04), antenatal care four and above (AOR = 1.6; 95% CI 1.22; 2.19), place of delivery (AOR = 9.67; 7.44, 12.57) and child is a twin (AOR = 3.33; 95% CI 1.23, 9.00) were variables significantly associated with newborn care.

**Conclusions:**

Newborn care practice in Ethiopia is below half per hundred participants. Even the distribution was not random. There is a need to pay attention to those cold spot areas and factors significantly associated with newborn care. Improving women’s educational levels secondary and above, and consolidating the continuation of antenatal care and health facility delivery were the priority areas to improve newborn care in Ethiopia. Maternal and neonatal health program managers and policymakers should pay attention to those cold spots of newborn care to achieve the sustainable development goal.

## Introduction

Maternal, neonatal, child and adolescent health is the priority area to achieve the sustainable development goal (SDG) [1]. Importantly, the neonatal period is one of the most crucial phases in the survival and development of a child. Most of the causes of neonatal mortality are preventable [2, 3]. Among the short list of causes of neonatal mortality preterm birth related complication accounts for 36%, intrapartum related event accounts for 26%, sepsis and other infectious diseases of newborn accounts for 8%, pneumonia accounts for 12%, and the remaining 20% of neonatal death due to diarrhea, tetanus, congenital anomalies, and other unspecified conditions [4, 5]. Therefore, most of the aforementioned causes of neonatal mortality can be tackled through preventive measures during pregnancy [6] and through consolidating simple, low-cost, and less time-consuming essential newborn care in the immediate post-partum period within the time limit of 2 days [7]. Every newborn everywhere has the right to get high-quality universal essential newborn health care.

Essential newborn care (ENC) is defined as a strategic approach planned to improve the health of new-born through intervention immediately after birth and during the rest of the postnatal period [8]. Essential newborn care involves immediate care at the time of birth, and during the entire newborn period. Essential newborn care can take place both in the health facility and at home. Because Essential newborn care (ENC) is a set of benchmarks for every neonate warrants regardless of where it is born. Essential newborn care is a cost-effective intervention that improves both maternal and neonatal health through timely averting the underlined cause as well as improving their nutritional status. Immediate care at birth includes delayed cord clamping, thorough drying, assessment of breathing, skin-to-skin contact, and early initiation of breastfeeding. Broadly newborn care practice is categorized as thermal care, assessment of health problems, resuscitation when needed, recognition and response to danger signs, support for breast milk feeding, nurturing care, infection prevention, and timely and safe referral when needed.

Evidence showed that improving the quality of newborn care is a cost-effective strategic approach to reducing early and late neonatal loss. A study done in India showed that the effect of collaborative quality improvement on reduction neonatal mortality at age 7 days (early neonatal period) is a difference in difference − 1.6, or 28 days is a difference in difference − 3.0 [9]. According to the American college of obstetrics and gynecology (ACOG) delayed umbilical cord clamping appears to be beneficial for term and preterm infants. Importantly, delayed umbilical cord clamping in term baby significantly associated with improved iron storage in the first several months of life [10]. Another important variable for essential newborn care is the initiation of breastfeeding. Evidence showed that if women did not breastfeed within 1 hour after birth, the odds of neonatal deaths increased by nearly three times as compared with those neonates who breastfed within 1 hour after birth [11]. After all, proven and cost-effective newborn survival interventions, such as early detection of pregnancy complications, clean delivery practices, early detection of danger signs, exclusive breastfeeding, and on-time diagnosis and treatment of neonatal complications like birth asphyxia and sepsis very crucial approach to avert the current neonatal death rate.

Though plenty of literature done about the prevalence and associated factors of newborn care practice (48.77% [12], 31% [13], and 24% [8]), there is a dearth of evidence about the spatial distribution of newborn care practice in Ethiopia. Newborn care practices are highly affected by the culture, beliefs, and values of the given society [14]. Developing countries are rich in cultural practices for the healing of diseases and care of mothers and their newborns [15, 16]. A mother who has just delivered her baby is considered as entering a ‘cold’ period and she strongly needs to have adequate rest after delivery and early clinic appointment including newborn care given commonly not routinely followed. If the healthcare worker visits her at her house, the family will appreciate it. However, they will not bring the mother or baby out of the house during the ‘cold’ period [17]. Based on this concept the authors hypothesized that “newborn care practice varies across regions of Ethiopia.” This study can give a clue about spatial projection, spatial autocorrelation, spatial interpolation or prediction, and spatial window of newborn care in Ethiopia for readers. Therefore, the study aimed to assess the spatial distribution and determinants of newborn care in Ethiopia.

## Methods

### Data and data source

We used secondary data from the Ethiopian Demographic and Health Survey (EDHS) conducted from the 18^th^ of January 2016 to the 27^th^ of June 2016. A cross-sectional survey was conducted in all regions of Ethiopia (Tigray, Afar, Amhara, Oromia, Somali, Benishangul-Gumuz, Southern Nations, Nationalities and Peoples Region (SNNPR), Gambella and Harari and two administrative councils–Addis Ababa and Dire Dawa). The total population of Ethiopia is 120 million and 80% of this huge number live in the rural part of the nation.

### Sample size determination and sampling procedure

Ethiopian Demographic and health survey 2016 data were used for the analysis of this study and the detail of the sampling procedure can be accessed from the report of the measure DHS website (www.dhsprogram.com). For this study, the women’s fill (IR) file was used to include women within two years postpartum period preceding the survey. The study population was determined by using the most recent live births of interviewed mothers in the 2 years preceding the survey. This means keeping the variable midx = 1 and keeping the variable b19<24 by using the STATA command gives the weighted sample size of 2796.

### Inclusion and exclusion criteria

All Postpartum period women with recent birth were included whereas those women who have no antenatal care and greater than 2 years’ post-partum period were excluded from the study.

### Variable of the study

#### Dependent variable

*Newborn care practice within two days of the postpartum period (Yes/No)*. It is a composite variable measured by six components cord examined (m78a = 1), the temperature measured (m78b = 1), counseling on danger signs (m78c = 1), counseling on breastfeeding (m78d = 1), observation of breastfeeding (m78e = 1) and weighed (m19a = 1 or 2) [18]. Then the outcome variable was dichotomized into participants whose newborn lacks one of the six components considered as not having newborn care and participants with all the six components considered as having newborn care practice within two days of the post-partum period.

*Independent variables*. independent variables were categorized into three catalogs of socio-demographic related factors (age of women, educational level, marital status, religion, media exposure, number of under-five children per household, wealth status, community educational level, community media exposure level, and community poverty level), maternal health and health facility related factors (timing of antenatal care start, number of antenatal care, antenatal care at the government health center, and place of delivery), and obstetrical and reproductive health-related factors (last child was wanted, history of pregnancy termination, the child was twin, sex of the child, and fertility preference “**Table 1**”.

**Table 1:** Operational definitions of independent variables.

Variables	Operational definition
Age	Age was a continuous variable at the beginning and now recoded as 15–20 = 1, 21–24 = 2 and 35–49 = 3 [19]
Marital status	Recoded never in union, living with partner, widowed, divorced, and no longer living together/separated = not currently married and married has kept as it is.
Wealth status	The variable was created wealth index combined poorer and poor = poor, middle = middle, and richer and rich = rich [20]
Religion	Catholic and traditional followed were categorized into “others” and the remaining kept as it is.
Number of under-five children per house hold	It was a continuous variable at the beginning and new recoded in to ≤1 and ≥2.
Media Exposure	It was created by combining the variable frequency of reading newspaper, frequency of listening radio, and frequency of visualizing television
Community women educational level	It was created form the variable v106 by the following steps first v106 recoded in to educated/yes and not educated/no, 2^nd^ tab v001the recoded v106 [iw = wt], 3^rd^ perform proportion by excel and save as csv coma delaminated, 4^th^ open new STATA and import it and save as STATA education, 5^th^ go to STATA data and combine the STATA by v001, 6^th^ codebook education, 7^th^ Recode <50% as community women educational level “low” and ≥50% as community women educational level “High”
Community media exposure level	The step as above and recoded as <50% = Low community media exposure level and ≥50% as high community media exposure level
Community poverty level	The step was as above and recoded as <50% = low community poverty level and ≥50% as high.
Timing of first antenatal care	It was a continuous variable and recoded as within first trimester and second trimester and above
Number of antenatal care visit	It was a continuous variable and recoded in to one up to two, three and four and above
Place of delivery	Generated by recoding other home, respondent home and other as “home delivery” and else as health facility delivery

### Data management and analysis

#### Spatial analysis

ArcGIS version 10.7 and **SaTScan version 9.6** statistical soft wares were used for exploring the spatial distribution/projection, global spatial autocorrelation, incremental autocorrelation, and spatial interpolation/prediction, for identifying significant hotspot areas of newborn care.

#### Spatial autocorrelation analysis

The spatial autocorrelation (Global Moran’s I) is the correlation coefficient for the relationship between a variable and its surrounding value, it measures the overall spatial autocorrelation of newborn care [21]. It measures whether newborn care practices are clustered, dispersed, or randomly distributed in the study area. Moran’s I is a spatial statistics measure used to measure spatial autocorrelation by taking the entire data set and producing a single output value that ranges from −1 to +1. The spatial autocorrelation coefficient is statistically significant when tested against the null hypothesis that the observed value differs from its expected value which is -1/ (n-1), where n is the number of points at the enumeration area level for which the autocorrelation is being computed. Moran’s I value ranges from -1 to 1 [22]. When Moran’s I values close to −1 indicate the outcome is dispersed/ shows a strong negative spatial autocorrelation, whereas I close to +1indicates the outcome of interest clustered/ shows a strong positive spatial autocorrelation and the outcome of interest distributed randomly/ there is no spatial autocorrelation if I value is zero. A statistically significant Moran’s I (*p* < 0.05) leads to the rejection of the null hypothesis (newborn care is randomly distributed) and indicates the presence of spatial autocorrelation [24].

#### Hot spot analysis (Getis-Ord Gi* statistics)

Getis-Ord Gi* statistics were computed to measure how spatial autocorrelation varies over the study location by calculating GI* statistic for each area. Z-score was computed to determine the statistical significance of clustering, and the *p*-value was computed for the significance. Statistical output with high GI* indicates a “hot spot” whereas low GI* means a “cold spot.”

#### Spatial interpolation/spatial prediction

The technique was used to predict newborn care in the unsampled areas in Ethiopia based on sampled clusters/enumeration areas (EAs). There are different geostatistical and deterministic interpolation methods. Of those various methods/techniques, empirical Bayesian Kriging and ordinary Kriging are the best interpolation techniques because they optimize the weight [23]. The ordinary Kriging spatial interpolation technique was used in the current study to predict newborn care in unobserved areas because it had a small residual and mean square error.

#### Spatial scan statistical analysis

In the spatial scan statistical analysis, a Bernoulli-based model was used in which events at particular places were analyzed, whether there is newborn care or not as 1/0. The scan statistics developed by Kulldorff and SaTScan software version 9.6 were used to identify the presence of purely spatial newborn care clusters. Scan statistics did scan gradually across the space to identify the number of observed and expected observations inside the window at each location. The scanning window with the maximum likelihood was the high-performing cluster, and a *p*-value was assigned to this cluster. In this study women whose newborns had the care were taken as cases and those who did not were considered as controls to fit the Bernoulli model. The number of cases in each location had a Bernoulli distribution and the model required data for cases, controls, and geographic coordinates.

#### Mixed effect logistic regression analysis

In this study, both random and fixed effect analysis was performed and the analysis was done using STATA version 14 Software. We applied the sampling weight to keep the representativeness of the study population to the overall enumeration area. Descriptive statistics like frequency and percent were performed and presented by tables and graphs. Importantly, a multilevel multivariable logistic regression analysis was done by considering the hierarchal nature of the data. First, bivariate multilevel logistic regression analysis was performed to find the crude odds ratio at a 95% confidence interval, and those variables statistically significant at 0.2 were used in the multilevel multivariable logistic regression analysis. Lastly, multilevel multivariable logistic regression analysis was performed to estimate the adjusted odds ratio and random variation between clusters. Statistically significant variables at a *p*-value less than 0.05 were reported with their 95% confidence interval.

In the multilevel multivariable logistic regression model, fixed effect estimates measure the association between the odds of newborn care of individual- and community-level factors with a 95% confidence Interval. Intra cluster Correlation Coefficient (ICC) quantifies the degree of heterogeneity of newborn care between clusters. The proportion of change in variance (PCV) measures the proportion of the total observed individual variation that is attributable to the between-cluster variations [24]. The median odds ratio (MOR) measures the value between high and low-risk clusters (EAs).

## Ethical considerations

Since the study was a secondary data analysis of publically available survey data from the MEASURE DHS program, ethical approval and participant consent were not necessary for this particular study. We requested DHS Program and permission was granted to download and use the data for this study from http://www.dhsprogram.com. The Institution Review Board-approved procedures for DHS public-use datasets do not in any way allow respondents, households, or sample communities to be identified. There were no names of individuals or household addresses in the data file. The geographic identifiers only go down to the regional level (where regions are typically very large geographical areas encompassing several states/ provinces). Each enumeration area (Primary Sampling Unit) has a PSU number in the data file, but the PSU numbers do not have any labels to indicate their names or locations. In surveys that collect GIS coordinates in the field, the coordinates are only for the enumeration area (EA) as a whole, and not for individual households, and the measured coordinates are randomly displaced within a large geographic area so that specific enumeration areas cannot be identified.

## Results

### Socio-demographic characteristics of participants

A total of 2796 post-partum period women were included in the study and 1942.66(69.49%) of them were within the age group of 20–34. In this study1537.74 (51.43%) of them have no formal education and 2636.27 (94.28%) of the participants were married at the time of the survey. Moreover, 2311.45 (82.68%) were rural in residence, 1170.60 (41.87%) were rich in wealth status, and 1113.45 (39.83%) were orthodox. Among all participants 1527.59 (54.04%) were have two and above under five children per household, 1621.88 (58.01%) were have no media exposure, 1422.88 (50.89%) were in low community media exposure level, 1621.01 (57.98%) were in low community educational level and 1455.42 (52.06%) were in low community poverty level **“Table 2.”**

**Table 2:** Socio-demographic factors of participants in Ethiopia.

Variables	Frequency	Percent
**Age**		
15–20	390.30	13.96
20–34	1942.66	69.49
35–49	462.81	16.55
**Educational Status of women**		
Have no formal Education	1537.74	51.43
Primary	987.05	35.31
Secondary	249.71	8.93
Higher education	121.26	4.34
**Marital Status**		
Currently not married	159.51	5.71
Currently married	2636.27	94.29
**Residency**		
Urban	484.32	17.32
Rural	2311.45	82.68
**Wealth status**		
Poor	1043.94	37.34
Middle	581.23	20.79
Rich	1170.60	41.87
**Religion**		
Others	87.86	3.14
Protestant	573.69	20.52
Muslim	1020.77	36.51
Orthodox	1113.45	39.83
**Region**		
Tigray	292.24	10.45
Afar	23.76	0.85
Amhara	605.68	21.66
Oromia	996.10	35.63
Somali	83.09	2.97
Benishangul	32.83	1.17
SNNPR	623.14	22.29
Gambela	7.45	0.27
Harari	7.45	0.27
Addis Ababa	108.00	3.86
Dire Dawa	16.00	0.57
**Number of under five children per house hold**		
One	1268.18	45.36
Two and above	1527.59	54.64
**Media exposure**		
No	1621.88	58.01
Yes	1173.88	41.99
**Community women’s education level**		
Low	1621.01	57.98
High	1174.76	42.02
**Community media exposure level**		
Low	1422.88	50.89
High	1372.89	49.11
**Community poverty level**		
High	1340.35	47.94
Low	1455.42	52.06

Others = Catholic, traditional followers

### Maternal health and health facility related factors

Among all participants, 1898.12 (67.89%) of them initiate antenatal care in the second trimester and above and 1436.38 (51.38%) of them have four and above antenatal care visits. Furthermore, 1790.47 (64.04%) were antenatal care attained at the government health centers and 1398.32 (50.02%) of them had been delivered at the health facility **“Table 3.”**

**Table 3:** Maternal health and health facility related factors.

Variables	Frequency	Percent
**Timing of first antenatal care visit**		
Within first trimester	897.65	32.11
Second trimester and above	1898.12	67.89
**Number of antenatal care visit**		
One–two	590.47	21.12
Three	768.91	27.50
Four and above visit	1436.38	51.38
**Antenatal care in government health center**		
No	1005.30	35.96
Yes	1790.47	64.04
**Place of delivery**		
Home	1397.45	49.98
Health facility	1398.32	50.02

### Obstetrical and reproductive health related factors

Among all participants 2129.65 (76.17%) were last-child wanted then, 2570.01 (91.92%) had no history of pregnancy termination, and 2754 (98.52%) were single birth. Moreover, 1468.52 (52.53%) of their child sex were female, 2324.11 (83.13%) had less than 5 under-five children, and 1797.61 (64.30%) of them with fertility preference of having another “**Table 4.”**

**Table 4:** Obstetric and reproductive health related factors.

Variables	Frequency	Percent
**Last child wanted**		
Wanted then	2129.65	76.17
Wanted later	493.28	17.64
Wanted no more	172.83	6.18
**Ever had terminated pregnancy**		
No	2570.01	91.92
Yes	225.76	8.08
**Child is twin**		
Single birth	2754.27	98.52
Second of multiple	41.50	1.48
**Sex of child**		
Female	1468.53	52.53
Male	1327.24	47.47
**Number of living children**		
Less than five	2324.11	83.13
Grand multi parity	471.66	16.87
**Fertility preference**		
Have an other	1797.61	64.30
Undecided	153.96	5.51
No more	844.19	30.20
**Duration of pregnancy**		
Late preterm	45.63	1.63
Term	2750.14	98.37

### The prevalence of newborn care in Ethiopia secondary analysis of 2016 Ethiopian demographic and health data

**Fig 1:**
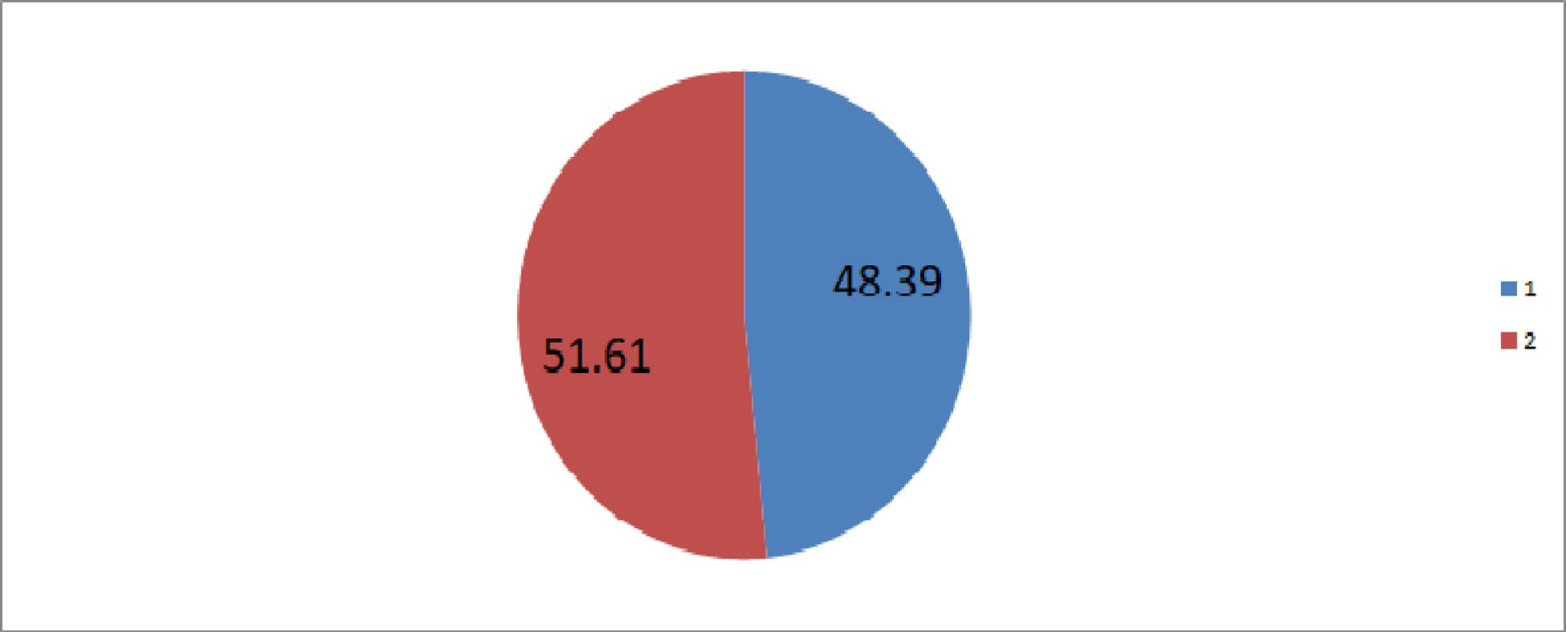
The prevalence of newborn care in Ethiopia, EDHS 2016 data. 1 = the proportion of newborn care and 2 = the proportion of not having newborn care.

In the current study the prevalence of newborn care was found to be 48.39% [95% CI 48.54%-50.24%] “**Fig 1”.**

### Multilevel logistic regression analysis of newborn care in Ethiopia, EDHS2016

In this study, both bivariate and multivariable analyses were done to detect statistically significant variables. In the bivariate analysis, a total of five individual-level variables (Educational status of women, wealth status, number of antenatal cares, place of delivery, and child is a twin) and three community-level variables (community women’s education level, community poverty level, and community media exposure level) were statistically significant with outcome variable newborn care. In multivariable multilevel analysis, a total of four variables (women’s educational status, number of antenatal cares, place of delivery, and child is a twin) were significantly associated with newborn care.

Women with secondary, and college and above educational level 1.67, and 2.47 times more likely their baby to have newborn care (AOR = 1.67; 95% CI 1.06, 2.62) and (AOR = 2.47; 95% CI 1.22, 5.01) as compared to women with have no formal education, respectively. Participants with the number of antenatal care three and four and above 1.50 and 1.60 times more likely to have newborn care (AOR = 1.50; 95% CI 1.10, 2.04) and (AOR = 1.60; 95% CI 1.22, 2.19) as compared to women with the number of antenatal cares one up to two, respectively.

Participants with the place of delivery at a health facility were 9.67 times more likely to have newborn care (AOR = 9.67; 95% CI 7.44, 12.57) as compared to women delivered at home. Women with second twin birth were 3.33 times more likely to have newborn care (AOR = 3.33; 95% CI 1.23, 9.00) as compared to the single birth “**Table 5”**.

**Table 5:** Multilevel logistic regression analysis of newborn care in Ethiopia, EDHS 2016.

Variable		Null model	Model I	Model II	Model III
			AOR[95%CI]	AOR[95%CI]	AOR[95%CI]
**Age of women**					
15–20			1		1
21–34			1.34[0.97,1.85]		1.32[0.95,1.82]
35–49			1.45[0.96,2.20]		1.43[0.94,2.16]
**Educational status**					
Have no formal education			1		1
Primary			0.97[0.75,1.26]		0.93[0.71,1.21]
Secondary			1.82[1.17,2.82]**		1.67[1.06,1.62]*
College and above			2.81[1.41,5.60]**		2.47[1.22,5.01]**
**First of antenatal care**					
Within first trimester			1.22[0.96,1.56]		1.21[0.95,1.55]
Second trimester and above			1		1
**Wealth status**					
Poor			1		1
Middle			1.08[0.80,1.48]		1.10[0.80,1.51]
Rich			1.38[1.02,1.87]*		1.34[0.96,1.85]
**Media exposure**					
No			1		1
Yes			1.09[0.85,1.41]		1.06[0.81,1.38]
**Number of antenatal care**					
One up to two			1		1
Three			1.5[1.10,2.05]***		1.5[1.10,2.04]**
Four and above			1.64[1.22,2.20]***		1.6[1.22,1.29]**
**Place of delivery**					
Home			1		1
Health facility			10.11[7.81,13.09]***		9.6[7.44,12.57]***
**Child is twin**					
First of multiple			1		1
Second of multiple			3.27[1.21,8.81]**		3.33[1.23,9.00]*
**Residency**					
Urban				1	**1**
Rural				0.13[0.08,0.21]	0.75[0.43,1.29]
**Community media exposure**					
Low				1	1
High				3.52[2.39,5.18]***	1.05[0.68,1.61]
**Community women education**					
Low				1	1
High				3.98[2.71,5.85]***	1.29[0.86,1.94]
**Community poverty level**					
Low				1	1
High				0.30[0.21,0.45]***	1.13[0.73,1.74]
**Model diagnosis**					
Log likely hood			-1395.71	-1612.1624	**-1393.8272**
Deviance			2791.42	3224.32	**2787.65**
**Random Effects**	**Null model**				
ICC	43.1[36.8,49.6]		31.6	35	31.6
PCV	Reference		65%	38%	65%
MOR	4.08		3.18	3.5	3.18

AOR: adjusted odds ratio, and

*. = 0.01≤ *p* value ≤ 0.05,

** = 0.001≤*p* value ≤0.01,

*** = *p* value = 000.

### Spatial distribution of newborn care in Ethiopia

**Fig 2:**
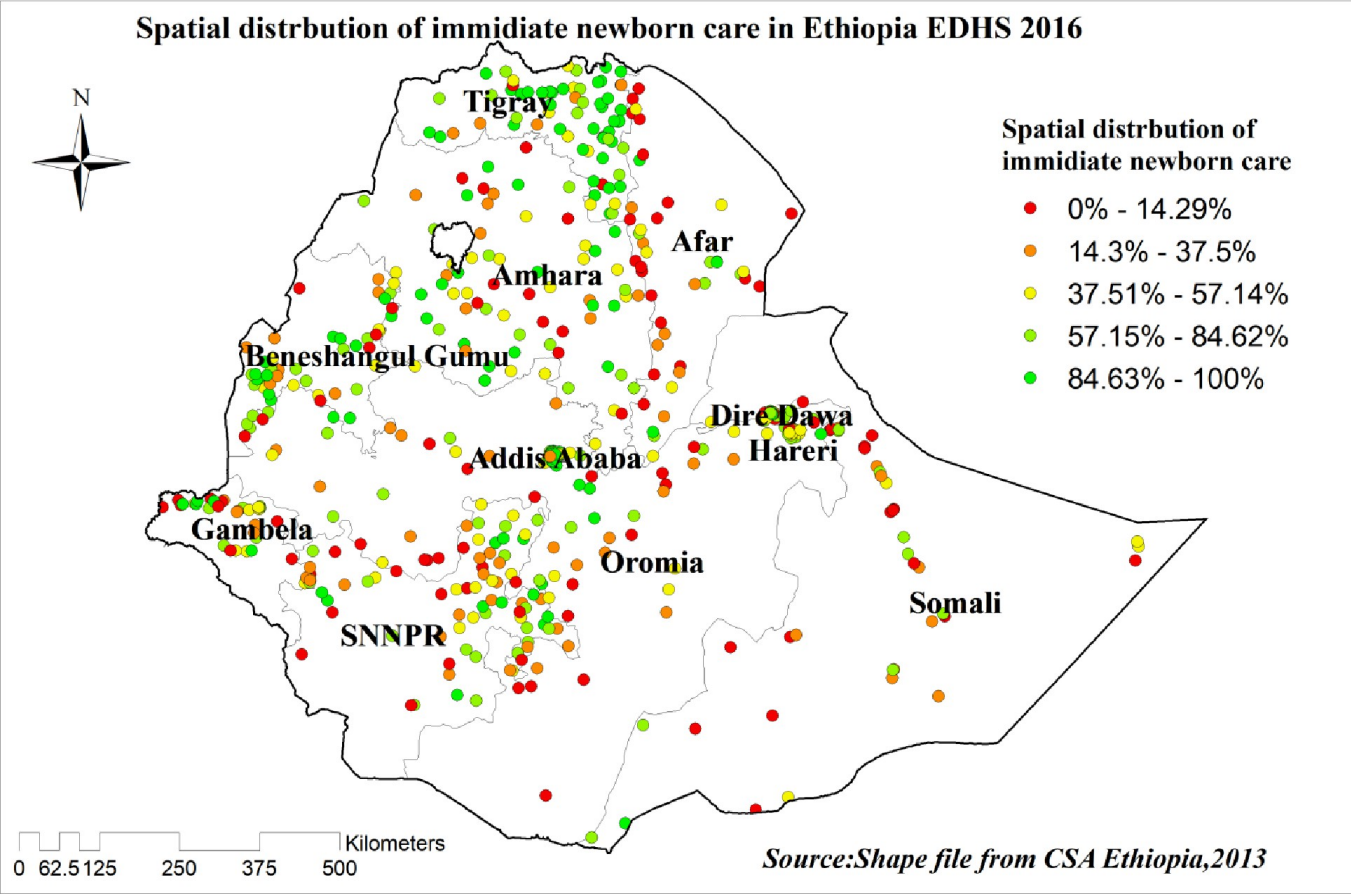
Spatial distribution/projection of newborn care in Ethiopia, EDHS 2016.

A total of 612 clusters were considered for the spatial analysis of newborn care in Ethiopia. On the map, each point represents one enumeration area with the proportion of newborn care in each cluster. The green color of the point on the map represents a high proportion of newborn care in the enumeration area whereas the red color indicates an enumeration area with a low proportion of newborn care. A higher proportion of newborn care was found in Tigray, Amhara, Benishangul Gumuze, and Addis Ababa. On the other hand, Somali and Oromia were characterized by a low proportion of newborn care within two days **“Fig 2”.**

### Spatial and incremental autocorrelation of newborn care in Ethiopia

**Fig 3:**
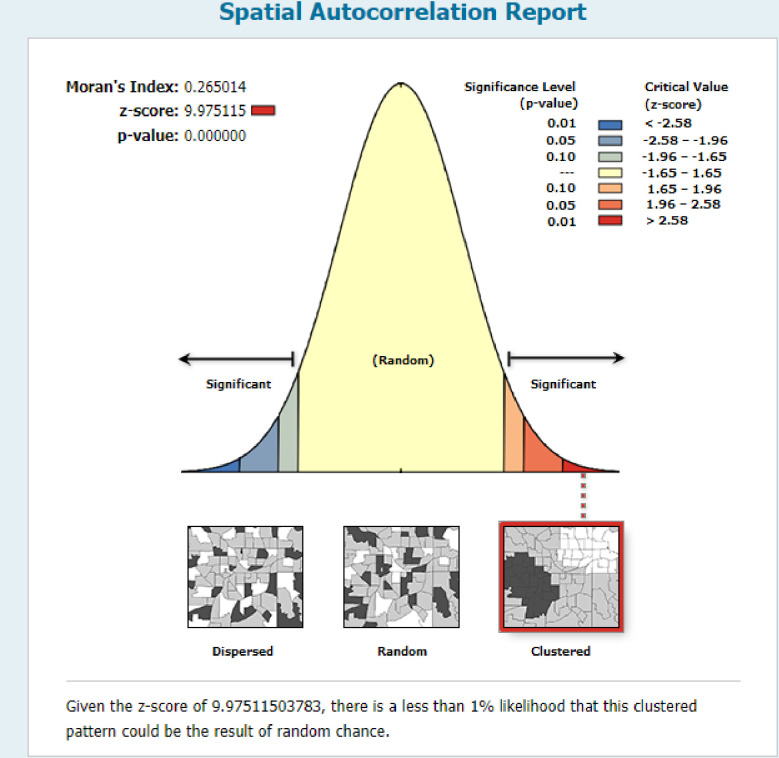
Spatial autocorrelation analysis of newborn care practice in Ethiopia, EDHS 2016.

**Fig 4:**
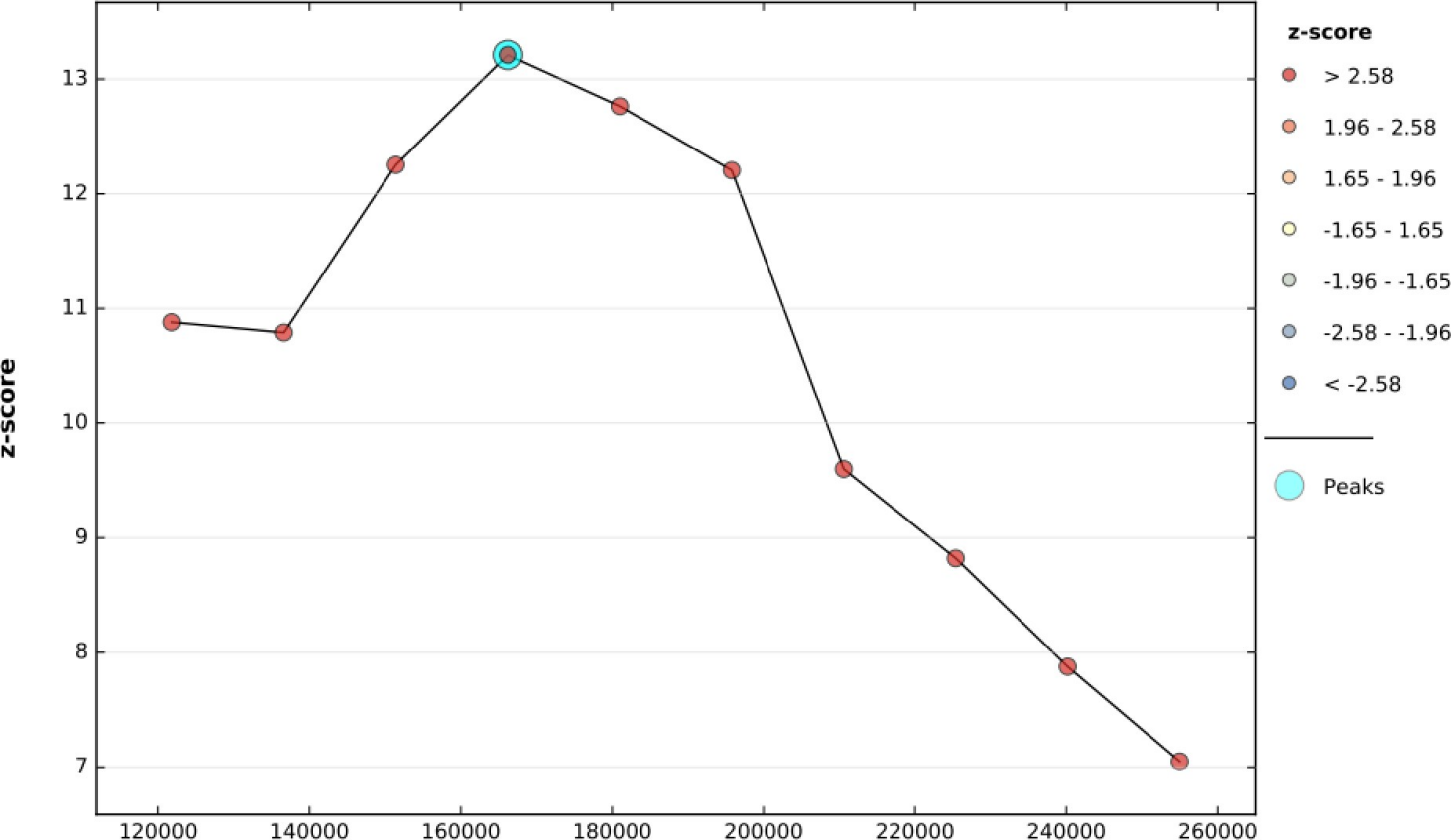
Incremental autocorrelation of newborn care practice in Ethiopia, EDHS 2016.

The clustered patterns (on the right sides) showed that a high rate of newborn care was observed. The *z* value showed that there is a clustered pattern with the probability of a chance <1%. The bright red and blue colors to the end tails indicated that there is an increased significance level **“Fig 3”.** The incremental spatial autocorrelation for a series of distances presented by a line graph with corresponding z-score was done to determine the average nearest neighbor and minimum and maximum distance band. A total of 10 distance bands were detected by a beginning distance of meters, 121814.96, and the first maximum peak (clustering) was observed at 166205.10 meters “**Fig 4”**.

### Hot spot analysis of newborn care

**Fig 5:**
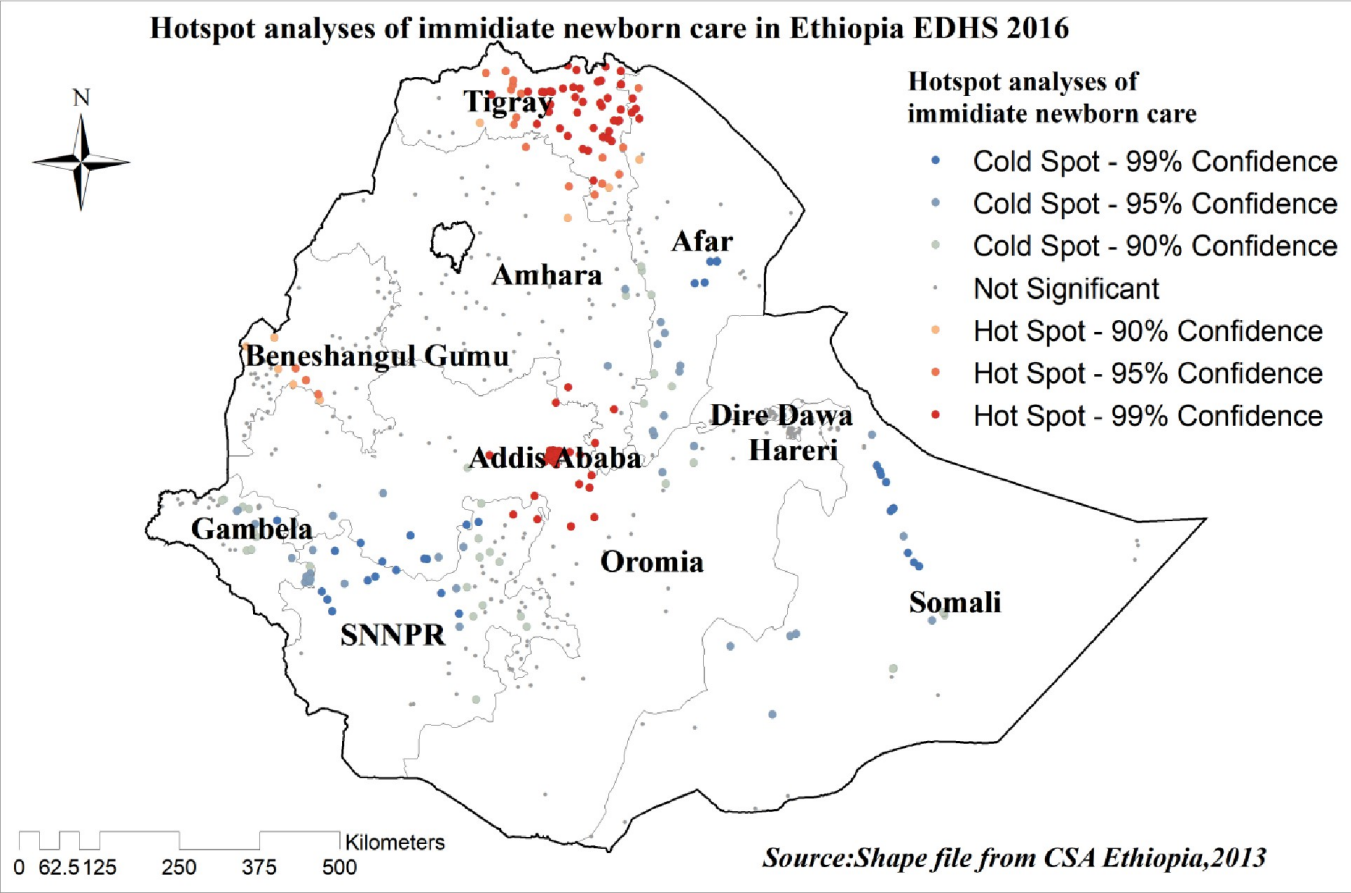
Hot spot analysis of newborn care in Ethiopia, EDHS 2016.

Hot spot analysis was done to identify areas with a high probability for newborn care practice. The red color indicates areas with a high probability of newborn care practice like Tigray, Benishangul Gumuze, and Addis Ababa. On the other hand, the blue color indicates newborn care practice is less likely performed “**Fig 5”**.

### Spatial interpolation/spatial prediction of newborn care

**Fig 6:**
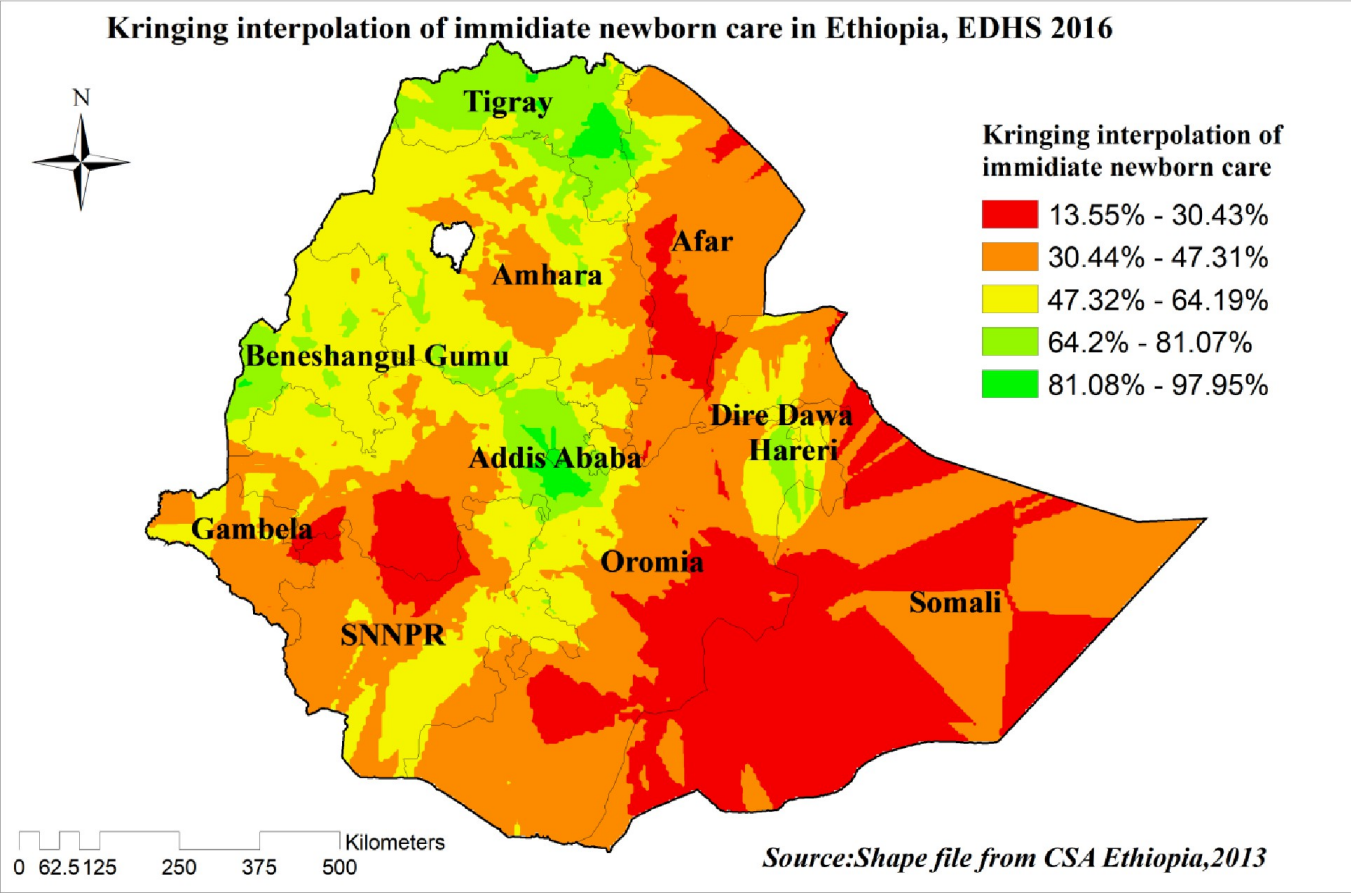
Spatial interpolation/prediction of newborn care in Ethiopia, EDHS 2016.

The possibility of newborn care practice was increasing while we move from the red- to the green-colored areas. The red and semi-red color predicts less possibility of newborn care practice and the green color predicts high possibilities areas of newborn care. Tigray, North West Addis Ababa, Harari, and Southeast Benishangul Gumuze were areas of highly predicted practice of newborn care. On the other hand, Somali, southwest of Oromia, south-central part of Afar, southeast of Addis Ababa, and northern part of SNNPR predicted fewer possibilities for newborn care practice “**Fig 6”**.

### Spatial SaTScan statistics analysis of newborn care in Ethiopia

**Fig 7:**
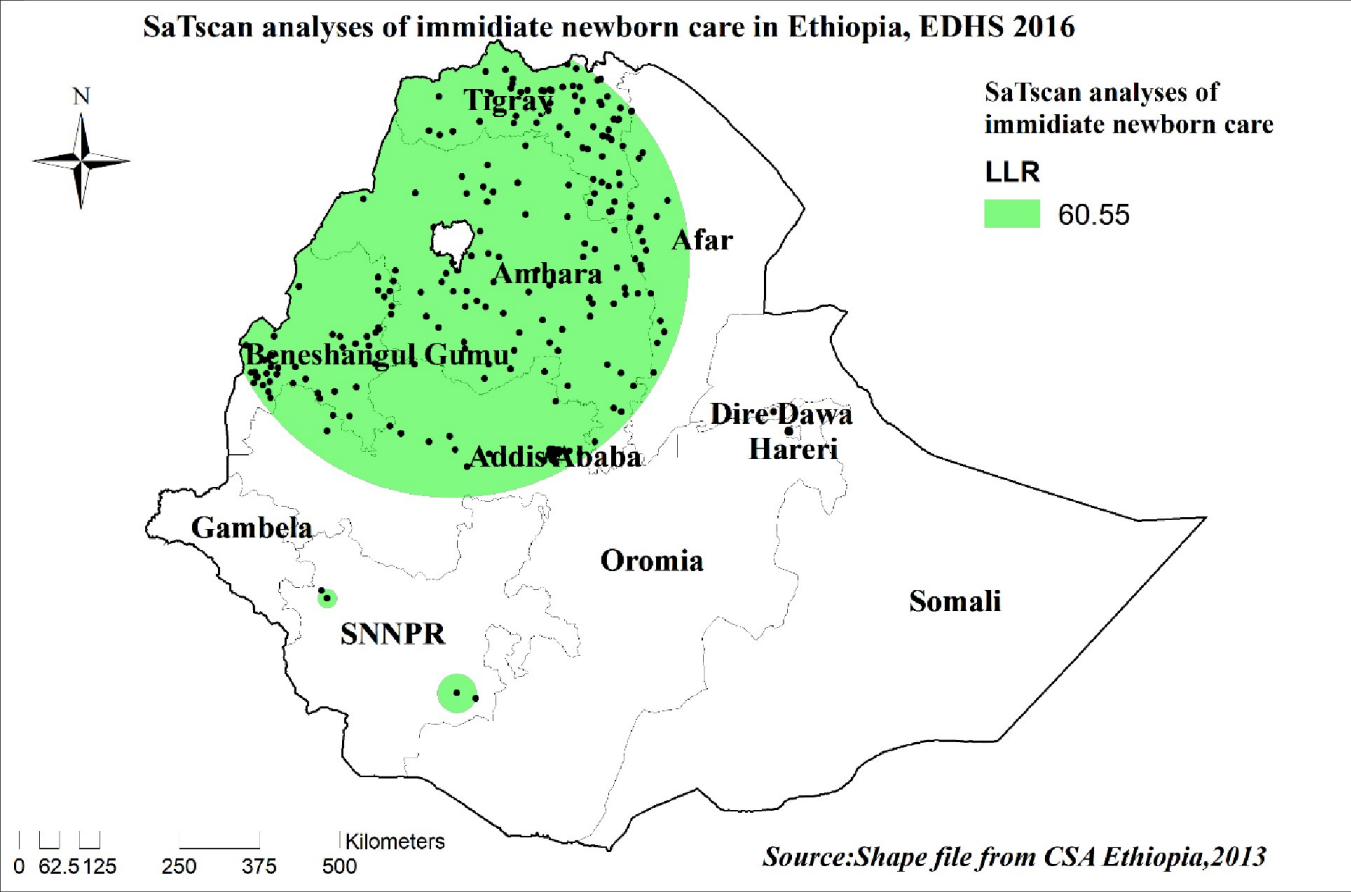
Significant clusters of newborn care spatial window in Ethiopia, EDHS 2016 plotted using ArcMap 10.7.

There were primary clusters of **newborn care** among reproductive-age women in Ethiopia. Of the total clusters, 257 were significant primary clusters. These were in the entire Amhara, Tigray, and most of Afar regions centered at 11.699828 N, 37.313042 E with a 368.36 km radius. Reproductive-age women who were found in the SaTScan window were 1.5 times more likely to have **newborn care** (RR = 1.48, P-value<0.0001) **“Table 6 and Fig 7”.**

**Table 6:** Significant spatial clusters of newborn care in Ethiopia, EDHS 2016.

Clusters	Enumeration areas (clusters) detected	Coordinate/radius	Population	Cases	RR	LLR	P-value
1* [257]	169, 73, 431, 158, 516, 382, 167, 512, 292, 163, 361, 456, 403, 429,132, 24, 259, 109, 602, 3, 541, 327, 640, 120, 515, 415, 548, 279, 386, 498, 375, 152, 38, 312, 627, 638, 199, 474, 206, 533, 246, 545, 628, 322, 559, 176, 482, 531, 52, 494, 36, 150, 218, 350, 66, 10, 183, 184, 296, 460, 591, 612, 401, 504, 137, 267, 425, 244, 542, 35, 354, 478, 510, 258, 616, 617, 300, 188, 256, 320, 136, 340, 392, 551, 496, 97, 455, 18, 253, 351, 143, 572, 575, 442, 579, 449, 538, 423, 611, 583, 399, 88, 345, 161, 424, 294, 156, 280, 189, 249, 160, 181, 98, 128, 70, 310, 488, 636, 65, 124, 254, 571, 584, 332, 234, 569, 79, 191, 255, 621, 344, 241, 457, 389, 597, 528, 268, 349, 400, 430, 355, 485, 237, 550, 94, 590, 81, 605, 335, 368, 384, 78, 637, 324, 209, 220, 118, 421, 481, 84, 563, 604, 544, 407, 304, 409, 274, 463, 112, 532, 11, 144, 339, 130, 369, 107, 626, 464, 31, 170, 45, 100, 108, 153, 305, 635, 582, 414, 314, 247, 487, 195, 547, 59, 623, 608, 645, 145, 287, 595, 639, 159, 461, 129, 428, 509, 560, 19, 293, 201, 433, 155, 90, 110, 99, 226, 225, 402, 264, 147, 61, 330, 211, 295, 172, 298, 620, 451, 539, 261, 475, 285, 303, 276, 599, 252, 416, 236, 203, 6, 581, 624, 17, 83, 317, 353, 341, 196, 508, 102, 121, 479, 40, 89, 165, 598, 127, 404	11.699828 N, 37.313042 E / 368.36 km	1198	778	1.48	60.55	<0.0001

* = significant primary clusters

## Discussion

Newborn care is one of the priority areas to achieve the sustainable development goal related to neonatal health [25]. Currently, newborn care is consolidated to be implemented even at the community level [26, 27]. But there is an inadequacy of evidence about the spatial variation of newborn care practice in Ethiopia. Therefore, the current study can be an input to show the spatial distribution of newborn care for further intervention by the responsible bodies.

In this study, the prevalence of newborn care is 48.39%. The finding of the current study by far higher than the previous study done in eastern Uganda at 11% [28]. On the other hand, the prevalence of immediate newborn care practice in the current study is lower than the study conducted in Midwest Nepal which was 58.6% [29]. The discrepancy in these findings might be attributed to the difference in methods used and study settings, socio-demographic characteristics of the study participants, and availability and accessibility of health service infrastructure. In the context of our locality, the finding is like evidence from a systematic review conducted in Ethiopia 48.77% [12]. The similarity might be due to the study’s representativeness of national-level evidence. On the other hand, slightly higher than the previous study done at Dessie referral hospital at 46.9% [30], Western Ethiopia at 44.1% [31], southwest Ethiopia at 41% [32], and a community-based study in Ethiopia at 44% [33]. The variation might be due to the difference in the study population which means women who had delivered at home mostly lacked contact with obstetric care providers and were limited from practicing newborn care.

The prevalence of newborn care is by far lower than the previous study conducted in the North Shoa administrative zone 73.8% [34], Sidama regional state 56.6% [35]. The possible explanation might be due to the difference in outcome measurement. This means in the previous study newborn care practice was considered when 70% of the components were fulfilled whereas the current study measures the outcome variable when all components were fulfilled and this might make a lower prevalence compared to the previous one. In addition, the current study was lower than the evidence done in the Awi zone where the prevalence of immediate newborn care practice was 62.7% [36]. The difference might be to the variation of the study population which means the current study is based on post-partum women so a woman might forget all the components of newborn care whereas the previous study was based on obstetric care providers and they might respond to all the components as they are professionally linked.

The spatial distribution of newborn care is not random and there is variation across Ethiopia. Among all regions of Ethiopia Tigray, Amhara, Benishangul Gumuze, and Addis Ababa are characterized by the high practice of newborn care. The variation might be imposed by the difference in socio-demographic status and due to the variation in the maternity continuum of care [37]. One of the elements in the maternity continuum of care is health facility delivery which is highly practiced in Tigray compared to the rest of the regions and this might inflate the prevalence of newborn care. Place of delivery is one factor statistically significant in the current study.

Regarding determinant factors educational status of women, the number of antenatal cares, place of delivery, and the child is twin are statistically significant with the outcome variable newborn care.

Women with the educational status of secondary and college and above are 1.67 and 2.47 times more likely to practice newborn care for their baby as compared to those women with no formal education respectively. The possible explanation is because of education is the engine of attending maternal and neonatal health care. Evidence showed that as educational level increases the probability of attending maternity continuity of care increases and then the proportion of newborn care practice might increase.

Participants with a number of antenatal cares three and four and above 1.5 and 1.6 times more likely their newborn has care as compared to women with antenatal care visit one up to two, respectively. A possible explanation could be due to antenatal care is a noteworthy predictor of subsequent maternity and newborn care. The number of antenatal care increases the contact rate with health care providers will increase and counseling about newborn care might increase and then newborn care practice might also increase.

Delivery at the health facility contributes 9.6 times higher newborn care as compared to participants delivered at home. The possible explanation could be that health facility delivery is one factor to access skilled maternity care providers and their newborn might have a chance to essential care.

Participants whose child is second of multiple 3.33 times more likely newborn care compared to participants with a single birth. The possible explanation could be that twin delivery is mostly associated with caesarian birth and this might lead to prolonged stay at a health facility and their newborn might have a chance of newborn care within two days.

## Strengths and limitations of the study

The main strength of this study was the use of weighted nationally representative data with a large sample which makes it representative at national and regional levels. Moreover, the use of a multilevel model took into account the hierarchical nature of the EDHS data and the variability within the community to get a reliable estimate and standard errors. But it is not free of limitations mainly resulting from the use of secondary data. As some important confounders like the health service quality and behavioral factors are missed. The other limitation of this study is considering women’s postpartum period 2 years prior to the survey might introduce recall bias.

## Conclusions

In the current study, the spatial distribution of newborn care practice in Ethiopia is not random and the national level prevalence is too low to achieve the sustainable development goal of neonatal mortality reduction. Educational level secondary, college and above, number of antenatal care three, and four and above, health facility delivery, and baby second of multiple births are positively associated with newborn care practices in Ethiopia. Program managers, policymakers, and planners of maternal and newborn health-related issues might take the finding of this study as an impute to directly act on the identified areas of cold spots in newborn care practice in Ethiopia.

## Supporting information

S1 File Data. (DTA)

## Abbreviations/acronyms

ACOG: American College of Obstetrics and Gynecology
AOR: Adjusted Odds Ratio
ICC: Interclass Correlation Coefficient
MOR: Median Odds Ratio
PCV: Proportion Change in Variance
SDG: Sustainable Development Goal

## References

[1] Das T., et al., Sustainable Development Goal 3: Good Health and Well-being, in South-East Asia Eye Health. 2021, Springer. p. 61–78.

[2] Sharrow D., et al., Global, regional, and national trends in under-5 mortality between 1990 and 2019 with scenario-based projections until 2030: a systematic analysis by the UN Inter-agency Group for Child Mortality Estimation. The Lancet Global Health, 2022. 10(2): p. e195–e206. 35063111 10.1016/S2214-109X(21)00515-5 PMC8789561

[3] Zelalem Ayichew M., et al., Neonatal mortality and associated factors among neonates admitted to neonatal intensive care unit of Gandhi memorial hospital in Addis Ababa, Ethiopia, 2019. BMC pediatrics, 2022. 22(1): p. 1–9. 35550058 10.1186/s12887-022-03339-6 PMC9097131

[4] Osman M.O., et al., Prevalence and causes of neonatal mortality among neonates admitted in neonatal intensive care unit at Sultan Hassan Yabare Referral Hospital, East Ethiopia 2019. Science, 2020. 9(1): p. 11–17.

[5] Muhe L.M., et al., Major causes of death in preterm infants in selected hospitals in Ethiopia (SIP): a prospective, cross-sectional, observational study. The Lancet Global Health, 2019. 7(8): p. e1130–e1138. doi: 10.1016/S2214-109X(19)30220-7 31303299 PMC6639243

[6] Organization, W.H., WHO recommendations on antenatal care for a positive pregnancy experience. 2016: World Health Organization. 28079998

[7] Oladapo O., et al., WHO model of intrapartum care for a positive childbirth experience: transforming care of women and babies for improved health and wellbeing. Bjog, 2018. 125(8): p. 918. doi: 10.1111/1471-0528.15237 29637727 PMC6033015

[8] Chichiabellu T.Y., et al., Essential newborn care practices and associated factors among home delivered mothers in Damot pulasa Woreda, southern Ethiopia. Reproductive health, 2018. 15(1): p. 1–11. 30261886 10.1186/s12978-018-0609-1 PMC6161384

[9] Zamboni K., et al., Effect of collaborative quality improvement on stillbirths, neonatal mortality and newborn care practices in hospitals of Telangana and Andhra Pradesh, India: evidence from a quasi-experimental mixed-methods study. Implementation Science, 2021. 16(1): p. 1–17. 33413504 10.1186/s13012-020-01058-z PMC7788546

[10] Care P.P., ACOG committee opinion. Obstetrics Gynecol, 2019. 134: p. e84–9. 10.1097/AOG.0000000000003425 31441826

[11] Phukan D., Ranjan M., and Dwivedi L., Impact of timing of breastfeeding initiation on neonatal mortality in India. International breastfeeding journal, 2018. 13(1): p. 1–10. 29988694 10.1186/s13006-018-0162-0 PMC6029033

[12] Alamneh Y., et al., Essential newborn care utilization and associated factors in Ethiopia: a systematic review and meta-analysis. BMC pregnancy and childbirth, 2020. 20(1): p. 1–9. doi: 10.1186/s12884-020-2804-7 32093648 PMC7041192

[13] Sakelo A.N., et al., Newborn care practice and associated factors among mothers of one-month-old infants in Southwest Ethiopia. International Journal of Pediatrics, 2020. 2020. 10.1155/2020/3897427 PMC7593759 33133199

[14] Kassahun G., Wakgari N., and Abrham R., Patterns and predictive factors of unhealthy practice among mothers during pregnancy, childbirth, postnatal and newborn care in Southern Ethiopia: a community based cross-sectional study. BMC research notes, 2019. 12(1): p. 1–6. 31533813 10.1186/s13104-019-4631-3 PMC6751892

[15] Sutan R. and Berkat S., Does cultural practice affects neonatal survival-a case control study among low birth weight babies in Aceh Province, Indonesia. BMC pregnancy and childbirth, 2014. 14(1): p. 1–13. doi: 10.1186/1471-2393-14-342 25269390 PMC4262197

[16] Premji S., et al., Sociocultural influences on newborn health in the first 6 weeks of life: qualitative study in a fishing village in Karachi, Pakistan. BMC pregnancy and childbirth, 2014. 14(1): p. 1–12. 25030836 10.1186/1471-2393-14-232 PMC4223389

[17] Hishamshah M., et al., Belief and practices of traditional post partum care among a rural community in Penang Malaysia. The Internet Journal of Third World Medicine, 2010. 9(2): p. 1–9.

[18] Croft T.N., Marshall A.M., and Allen C.K., Guide to DHS Statistics. DHS-7: The Demographic and Health Surveys Program. ICF, Rockville, 2018.

[19] Asratie M.H., Kassie B.A., and Belay D.G., Prevalence of Contraceptive Non-use Due to Husbands/Partners Influence Among Married Women in Ethiopia: A Multilevel Analysis Using Demographic and Health Survey 2016 Data. Frontiers in Reproductive Health, 2022. 4: p. 876497. doi: 10.3389/frph.2022.876497 36303621 PMC9580793

[20] Asratie M.H. and Belay D.G., Pooled Prevalence and Determinants of Completion of Maternity Continuum of Care in Sub-Saharan Africa: A Multi-Country Analysis of Recent Demographic and Health Surveys. Frontiers in Global Women’s Health, 2022. 3. 10.3389/fgwh.2022.869552 PMC9174640 35692945

[21] Waldhör T., The spatial autocorrelation coefficient Moran’s I under heteroscedasticity. Statistics in Medicine, 1996. 15(7‐9): p. 887–892. doi: 10.1002/(sici)1097-0258(19960415)15:7/9<887::aid-sim257>3.0.co;2-e 8861157

[22] Chen Y., New approaches for calculating Moran’s index of spatial autocorrelation. PloS one, 2013. 8(7): p. e68336. doi: 10.1371/journal.pone.0068336 23874592 PMC3709922

[23] Bhunia G.S., Shit P.K., and Maiti R., Comparison of GIS-based interpolation methods for spatial distribution of soil organic carbon (SOC). Journal of the Saudi Society of Agricultural Sciences, 2018. 17(2): p. 114–126.

[24] Næss Ø. and Leyland A.H., Analysing the effect of area of residence over the life course in multilevel epidemiology. Scandinavian journal of public health, 2010. 38(5_suppl): p. 119–126. 21062846 10.1177/1403494810384646

[25] Paulson K.R., et al., Global, regional, and national progress towards Sustainable Development Goal 3.2 for neonatal and child health: all-cause and cause-specific mortality findings from the Global Burden of Disease Study 2019. The Lancet, 2021. 398(10303): p. 870–905. doi: 10.1016/S0140-6736(21)01207-1 34416195 PMC8429803

[26] Gogia S., et al., Community based newborn care: a systematic review and meta-analysis of evidence: UNICEF-PHFI series on newborn and child health, India. Indian pediatrics, 2011. 48(7): p. 537–546. doi: 10.1007/s13312-011-0096-8 21813923

[27] Avan B.I., et al., Embedding Community-Based Newborn Care in the Ethiopian health system: lessons from a 4-year programme evaluation. Health policy and planning, 2021. 36(Supplement_1): p. i22–i32. doi: 10.1093/heapol/czab085 34849897 PMC8633669

[28] Owor M.O., et al., Factors associated with adoption of beneficial newborn care practices in rural eastern Uganda: a cross-sectional study. BMC pregnancy and childbirth, 2016. 16(1): p. 1–11. doi: 10.1186/s12884-016-0874-3 27101821 PMC4840909

[29] Sanjel K., et al., Patterns and determinants of essential neonatal care utilization among underprivileged ethnic groups in Midwest Nepal: a mixed method study. BMC pregnancy and childbirth, 2019. 19(1): p. 1–10. 31455264 10.1186/s12884-019-2465-6 PMC6712593

[30] Semanew Y., et al., Newborn care practices and its determinants among postnatal mothers in Dessie Referral Hospital, Northeast Ethiopia. BMC research notes, 2019. 12(1): p. 1–6. 30791953 10.1186/s13104-019-4133-3 PMC6385447

[31] Efa B.W., et al., Essential new-born care practices and associated factors among post natal mothers in Nekemte City, Western Ethiopia. PloS one, 2020. 15(4): p. e0231354. doi: 10.1371/journal.pone.0231354 32315342 PMC7173873

[32] Abebe H., Adane D., and Shitu S., Essential newborn care practice and its associated factors in Southwest Ethiopia. Archives of Public Health, 2021. 79(1): p. 1–9. 33789738 10.1186/s13690-021-00568-6 PMC8011223

[33] W/senbet M., et al., Community-based new born care practice and its associated factors among women who give birth at home in Ethiopia: cross-sectional study. Current Medical Research and Opinion, 2022. 38(3): p. 383–392. doi: 10.1080/03007995.2022.2026669 34994252

[34] Ashenef G., et al., Essential newborn care practice and associated factors among health care providers in Northeast Ethiopia: a cross-sectional study. Archives of Public Health, 2021. 79(1): p. 1–8. 34074312 10.1186/s13690-021-00613-4 PMC8167947

[35] Gonfa D.N., et al., Essential newborn care practice and associated factors among obstetric care providers of public hospitals in Sidama regional state, Ethiopia. SAGE open medicine, 2022. 10: p. 20503121221085840. doi: 10.1177/20503121221085840 35371485 PMC8972914

[36] Ayenew A., Abebe M., and Ewnetu M., Essential newborn care and associated factors among obstetrical care providers in awi zone health facilities, Northwest Ethiopia: an institutional-based cross-sectional study. Pediatric Health, Medicine and Therapeutics, 2020. 11: p. 449. doi: 10.2147/PHMT.S276698 33204205 PMC7667187

[37] Hunie Asratie M. and Belay D.G., Pooled Prevalence and Determinants of Completion of Maternity Continuum of Care in Sub-Saharan Africa: A Multi-Country Analysis of Recent Demographic and Health Surveys. Frontiers in Global Women’s Health, 2022: p. 56. doi: 10.3389/fgwh.2022.869552 35692945 PMC9174640

